# The Effect of Grapefruit Juice on the Pharmacokinetics of Tadalafil in Rats

**DOI:** 10.1155/2020/1631735

**Published:** 2020-01-24

**Authors:** Xiuwei Shen, Fan Chen, Fengwei Wang, Peng Huang, Wenchao Luo

**Affiliations:** Ruian People's Hospital, The Third Affiliated Hospital of Wenzhou Medical University, 325000 Wenzhou, China

## Abstract

We developed and validated a novel, sensitive, selective, and inexpensive high-performance liquid chromatography (HPLC) method for the determination of tadalafil in rats plasma and to investigate the effect of grapefruit juice on the pharmacokinetics of tadalafil in rats. The ZORBAX Eclipse XDB-C18 (4.6 × 150 mm, 5 *μ*m) chromatography column can be used to separate tadalafil and carbamazepine (internal standard, IS). A mixture of acetonitrile-0.2% trifluoroacetic acid-water (48 : 10 : 42, V/V/V) was used as the mobile phase with a flow rate of 1.0 mL/min. The column temperature was set at 35.0°C. The detection wavelength was set at 286 nm. The tadalafil was extracted by ethyl acetate from plasma at the alkaline condition. 12 healthy male Sprague-Dawley (SD) rats were randomly divided into two groups, Group A (experimental group, received grapefruit juice 5 mL/kg for 7 days) and Group B (control group, received normal saline for 7 days). All the rats were given a single dose of tadalafil (5 mg/kg) after the last administration. The main pharmacokinetic parameters were calculated by DAS 2.0 software. Under the conditions of this experiment, the plasma concentrations of tadalafil in the range of 10–2000 ng/ml had a good linear relationship. The intra- and interday precision for tadalafil in plasma were less than 15%, and the relative recovery rate was good at low, medium, and high QC levels. The *C*_max_ of tadalafil in the control group and the experimental group was (725.89 ± 161.59) ng/mL and (1271.60 ± 179.31) ng/mL, *t*_1/2_ was (9.28 ± 2.07) h and (11.70 ± 1.47) h, AUC _(0-*t*)_ was (7399.61 ± 696.85) ng·h/mL and (9586.52 ± 2048.81) ng·h/mL, and AUC_(0-∞)_ was (7995.50 ± 707.23) ng·h/mL and (10639.43 ± 2235.94) ng·h/mL, respectively. Results show that the *C*_max_ of tadalafil in group A was 75.17% higher than that in group B, the Vz/F was also reduced, and the *t*_1/2_ was increased by 2.42 h. The developed HPLC–DAD method for the determination of tadalafil in rats plasma was accurate, reproducible, specific, and it was found to be suitable for the pharmacokinetics of tadalafil and food-drug interactions. Grapefruit juice can inhibit the metabolism of tadalafil and increase the exposure of tadalafil in rats.

## 1. Introduction

Tadalafil is a competitive inhibitor of PDE-5, which prevents the degradation of cyclic guanosine monophosphate (cGMP), leading to an increase in cGMP and induction of corpus cavernosum smooth muscle relaxation, thereby increasing blood flow and causing penile erection [1, 2]. Tadalafil has been approved for pulmonary arterial hypertension (PAH) [3], erectile dysfunction (ED) [4], benign prostatic hyperplasia (BHP) [5], the lower urinary tract symptoms (LUTS) [6], and so on. Tadalafil was approved for the treatment of ED in 2009.

Tadalafil was rapidly absorbed after oral administration and reaches *C*_max_ 2 hours after taking the drug. The absorption rate and degree of tadalafil are not affected by food, so this product can be taken with or without food. Tadalafil is metabolized in the body primarily to inactive catechol metabolites by CYP3A4. Tadalafil is mainly excreted by feces, and about 1/3 of the metabolized drugs are excreted from the urine [7–9].

Tadalafil is mainly metabolized by CYP3A4. Studies have shown that some drugs inhibit CYP3A4 and increase exposure to tadalafil. Ketoconazole (400 mg/d) is a potent inhibitor of CYP3A4, which increases the AUC and *C*_max_ of tadalafil (20 mg/d) by 312% and 22%, respectively. Ketoconazole (200 mg/d) increased the AUC and *C*_max_ of tadalafil (10 mg/d) by 107% and 15%, respectively, compared to the single dose of tadalafil (10 mg). Although there are no specific interaction studies, other CYP3A4 inhibitors, such as erythromycin, itraconazole, and grapefruit juice may also increase the exposure levels of tadalafil [10, 11].

Grapefruit belongs to the family Rutaceae owing to several bioactive substances, such as flavonoids, carotenoids, coumarins, and organic acids, with a variety of health-promoting properties, such as anti-inflammatory, anticancer, and weight loss [12–14]. Flavonoids are seen as the most important bioactive components present in grapefruit. It has the functions of antioxidants, free radical elimination, tumor prevention, and cancer cell proliferation [15]. Numerous studies have shown that furocoumarin in grapefruit interacts with drugs by interfering with tactile and intestinal enzymes cytochrome P450 [16]. Studies have shown that both food and grapefruit juice can increase the exposure of blonanserin and N-desethyl blonanserin. Grapefruit juice improves bioavailability and may have reduced systemic clearance of blonanserin. The obvious explanation is that grapefruit juice can inhibit the activity of CYP3A4 in the intestinal [17].

Food-drug interactions can produce negative effects in the safety and efficacy of drug therapy, as well as in the nutritional status of the patient [18]. Tadalafil is metabolized by CYP3A4 in the body, and grapefruit juice is an inhibitor of CYP3A4. It may affect the metabolism of tadalafil in the body. A method for the determination of the concentration of tadalafil in rat plasma by HPLC was established. By measuring the concentration of tadalafil in the plasma and studying the effect of grapefruit juice on the pharmacokinetics of tadalafil in rats, it can provide a theoretical basis for food-drug interaction.

## 2. Experimental

### 2.1. Chemicals Materials

Tadalafil (purity > 98%, CAS: 171596-29-5) and carbamazepine (CBZ, purity > 98%, CAS: 100142–199503) were provided from Sigma (St. Louis, MO, USA). Tadalafil tablets (5 mg, CAS: C791509) were provided from Lilly del Caribe, Inc. Grapefruit juice (purity 100%) was obtained from the fresh fruit juice industry Kunshan co. LTD. (Kun Shang, China). Methanol and acetonitrile were of HPLC grade and were provided by Merck Company (Darmstadt, Germany).

### 2.2. Instrumentation and Conditions

The analyses were performed using an Agilent 1100 liquid chromatographic system equipped with a G1379A vacuum degasser, a G1311A quaternary pump, a G1316A column oven, a G1313A autosampler, and G1315B DAD detector.

Samples were separated on a ZORBAX Eclipse XDB-C18 (4.6 × 150 mm, 5 *μ*m, Agilent, USA) and XDB-C18 protection column at 35°C. The mobile phase consisted of acetonitrile-0.2% trifluoroacetic acid-water (48 : 10 : 42). All compounds were detected at an optimum wavelength of 286 nm, and the flow rate of the mobile phase was 1.0 mL/min.

### 2.3. Preparation of Standard and Quality Control (QC) Samples

A stock solution of 1 mg/mL was prepared by weighing 10 mg of tadalafil and dissolving it in 10 ml of methanol. The standard application solution was diluted with methanol to the concentrations of 100 *μ*g/mL, 10 *μ*g/mL, and 1 *μ*g/mL. A stock solution of 1 mg/mL was prepared by weighing 1 mg of CBZ and dissolving it in 1 ml of methanol and then diluting it to 4.0 *μ*g/mL standard solution. All of the solutions were stored in a refrigerator at 4°C. Calibration curve standards were prepared by adding appropriate amounts of the working solutions in blank rat plasma. The final concentrations of tadalafil were 10, 25, 50, 100, 250, 500, 1000, and 2000 ng/mL, respectively, and the concentration of IS was 400 ng/mL in rat plasma. The preparation of QC samples was the same, with three levels of plasma concentrations (25, 500, and 1500 ng/mL).

### 2.4. Sample Preparation

Before the analysis, the plasma samples were thawed to room temperature. In a 2 mL EP tube, an aliquot of 20 *μ*L of the IS working solution (4.0 *μ*g/mL) and 200 *μ*L of NaOH (1 mol/L) were added to 200 *μ*L of collected plasma sample followed by the addition of 1.0 mL ethyl acetate. The tubes were vortex mixed for 1.0 min and centrifuged at 3,000 rpm for 10 min at 4°C. Then, an aliquot of 900 *μ*L supernatant organic layer was transferred into a 1.5 mL EP tube and dried under a nitrogen stream at 40°C. The dried residue was reconstituted in 100 *μ*L of a mobile phase and a 10 *μ*L aliquot was injected into the HPLC system for the analysis.

### 2.5. Method Validation

To evaluate the selectivity of the method, the blank rats plasma and blank plasma spiked tadalafil and CBZ were analyzed. Calibration curves were constructed and validated by analyzing spiked calibration samples for three days in a row. The peak area ratio of tadalafil and CBZ was *y*, and the ratio of the concentration of tadalafil to CBZ was *x*, and standard curves were fitted by weighted (1/*χ*^2^) least squares linear regression in the concentration of 10–2000 ng/mL.

Accuracy and precision were assessed by the determination of QC samples at three concentration levels (25, 500, and 1500 ng/mL) in six replicates. On the same day, the intraprecisions were calculated, and the interprecisions were calculated by continuous measurement within 3 days.

The recoveries of tadalafil at three QC levels (25, 500, and 1500 ng/mL, *n* = 6) were determined by comparing the peak area of the analytes in the sample with the analyte added before extraction and the sample with the corresponding solution after extraction. The RSD of each concentration recovery should be within 15%.

The stabilities of tadalafil in rat plasma were tested by analyzing six replicates of plasma samples at three concentration levels (25, 500, and 1500 ng/mL) in different conditions. The short-term stability was determined after the exposure of the spiked samples at room temperature for 24 h. The freeze-thaw stability was evaluated after three complete freeze-thaw cycles (−20°C) on consecutive days. The long-term stability was assessed after storage of the standard spiked plasma samples at −20°C for 21 days.

### 2.6. Animals

Male Sprague-Dawley rats with body weights of 220 ± 20 g were obtained from Henan University of Science and Technology. The rats were adapted to the new environment for 7 days in laboratory conditions. Necessary approval from the Institutional Animal Ethics Committee was obtained to carry out the experiments.

### 2.7. Study Design

Twelve Sprague-Dawley male rats were randomly divided into 2 groups: Group A (experimental group, long-term administered with 5 ml/kg grapefruit juice for 7 days) and Group B (control group, the control group received normal saline for 7 days). All the rats were given a single dose of tadalafil with a concentration of 5 mg/kg. All the blood samples (0.3 mL) were collected from the tail-vein into heparinized 1.5 mL polythene tubes at 0.33, 0.67, 1, 1.5, 2, 3, 4, 6, 8, 12, 24, 36 and 48 h after tadalafil oral administration. The samples were immediately centrifuged at 10000 rmp for 10 min. The plasma obtained (200 *μ*L) was stored at −20°C until analysis.

### 2.8. Statistical Analysis

The mean and standard deviation (SD) were used for the results. The compartmental analysis was used to calculate the pharmacokinetic parameters by DAS 2.0 (Drug and statistics) software. The statistical analyses were evaluated by unpaired *t*-test (SPSS 19.0, Chicago, IL). A value of *p* < 0.05 was considered to be statistically significant.

## 3. Results

### 3.1. Sensitivity

**Figure 1:**
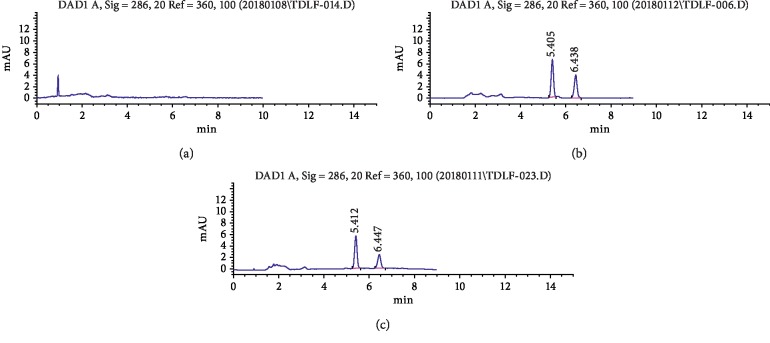
Representative HPLC for tadalafil and CBZ (IS) in rat plasma. (a) Blank plasma sample; (b) blank plasma sample spiked with tadalafil and CBZ; (c) rat plasma sample obtained 1.5 h after oral administration.

Under the experimental conditions described, tadalafil and CBZ were well separated from endogenous materials. Representative chromatograms of a blank plasma sample, a plasma sample spiked with tadalafil and CBZ, and a rat sample obtained 1.5 h after oral administration of tadalafil were shown in Figure 1. The retention time of tadalafil and CBZ were 6.41 and 5.44 min, respectively.

### 3.2. The Linearity of Calibration Curve

The linear regressions of the peak area ratios versus concentrations were fitted over the concentration range of 10–2000 ng/mL. The typical equations of the calibration curve were as follows: *y* = 0.0124*x* + 0.0638, *r* = 0.999.8. The LLOQ of tadalafil in rat plasma was 10 ng/mL.

### 3.3. Precision and Accuracy

The precision of the method was evaluated by calculating RSD for QCs at three concentration levels (25, 500, and 1500 ng/mL) over three validation days. The intraday RSDs were 4.69%, 3.27%, and 4.54% and the interday RSDs were 8.28%, 6.29%, and 4.86%, respectively, at three concentrations.

The accuracy of the method ranged from -2.00% to 3.88% for tadalafil at three QC levels. Assay performance data were presented in Table 1. The results demonstrated that the values were within the acceptable range and the method was accurate and precise.

### 3.4. Recovery

Mean extraction recoveries of tadalafil in rat plasma were (78.16 ± 3.14)%, (81.38 ± 1.93)%, and (80.56 ± 1.68)% (*n* = 6) at the concentrations of 25, 500, and 1500 ng/mL, respectively (Table 2).

### 3.5. Stability

The RSDs of the three quality control plasma samples (25, 500, and 1500 ng/mL) with spiked tadalafil were less than 10%, and tadalafil has shown good stability in plasma for 24 h at room temperature, during three freeze-thaw cycles, and for 21 days at −20°C (Table 3).

### 3.6. Effect of Grapefruit Juice on the Pharmacokinetic of Tadalafil

**Figure 2:**
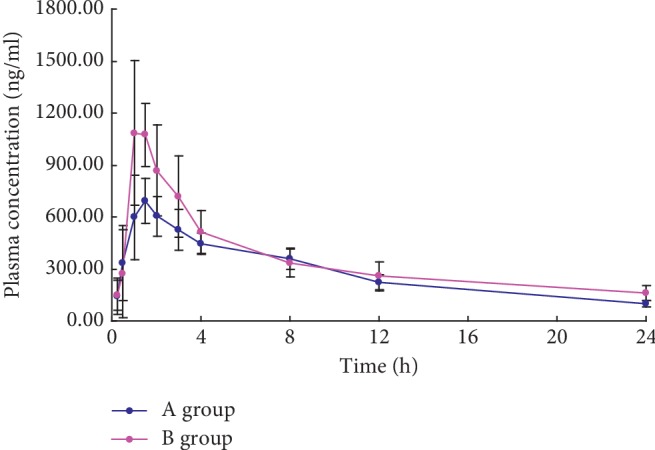
Mean plasma concentration time profiles of tadalafil in 2 groups after oral administration of 5 mgkg tadalafil (Means ± SD, *n* = 6).

Figure 2 reveals the mean plasma concentration time profiles of tadalafil after oral administration of tadalafil (5 mg/kg) in the different treatment group. The corresponding pharmacokinetic parameters are revealed in Table 4. As shown in Table 4 and Figure 2, grapefruit juice significantly altered the pharmacokinetic parameters of tadalafil. Compared with group A, the *C*_max_ of tadalafil was significantly increased by 57.4% by grapefruit juice. Moreover, the AUC_(0-∞)_ of tadalafil was increased by 29.55%. In addition, the Vz/F was also reduced, and the *t*_1/2_ was increased by 2.42 h. According to the data, it showed that grapefruit juice has a significant influence on the pharmacokinetics of tadalafil in rats.

## 4. Discussion

Food-drug interactions can produce negative effects in the safety and efficacy of drug therapy, as well as in the nutritional status of the patient [18]. The actual proportion of adverse drug reactions due to food-drug interactions is not known and, unfortunately, only when a serious adverse drug reaction follows a food-drug interaction does the matter receive significant attention [19].

Tadalafil is the substrate of CYP3A4, which is mainly metabolized by CYP3A4. Studies have shown that drugs that inhibit CYP3A4 can increase the exposure levels of tadalafil. Rifampicin (600 mg/day) is a CYP3A4 inducer, which can reduce the AUC and Cmax of tadalafil by 88% and 46%, respectively, compared with the 10 mg single dose of tadalafil [10, 11].

Grapefruit juice is a common inhibitor of CYP3A4 enzyme [20], which can slow down drug metabolism through liver metabolism, resulting in increased drug concentration in the body and increased adverse reactions. As the medications, such as darifenacin, fesoterodine, oxybutynin, and solifenacin, are metabolized in the liver by CYP3A4, their potential to interact with grapefruit juice cannot be neglected [21, 22].

In our experiment, we gave grapefruit juice to rats for 7 days, which had sufficient effects on enzymes and proteins in the body of rats. Tadalafil is the substrate of CYP3A4 and is mainly metabolized by CYP3A4. Compared with the control group, the *t*_l/2_ of tadalafil was significantly prolonged in the experimental group. Results show that the *C*_max_ of tadalafil in group A (experimental group) was 75% higher than that in group B (control group). The CLz/F was also reduced, and the half-life was increased by 2.42 h. In combination with grapefruit juice, the AUC and the half-life of tadalafil were increased, while grapefruit juice increased the exposure of tadalafil. These results suggest that grapefruit juice could inhibit the metabolism of tadalafil.

Although there are some differences in metabolism between different species, the results of animal experiments could provide a references for clinical medication. Therefore, when the grapefruit juice and tadalafil would be used together, the drug dose should be adjusted to ensure the efficacy and avoid adverse reactions. Specific medicinal substances, or supplements, consumed with food may significantly affect the efficacy and safety of the therapy. Gaps in knowledge on interactions especially with respect to the consequences of food-drug interactions are evident [23].

## 5. Conclusions

The HPLC–DAD method established in this study was used to detect tadalafil in rat plasma. It has high specificity, complete separation, and fast detection time. It was suitable for the pharmacokinetics and food-drug interaction studies of tadalafil. Grapefruit juice could increase the plasma exposure of tadalafil in the body, suggesting that grapefruit juice could inhibit the metabolism of tadalafil. Patients using tadalafil should pay attention to food-drug interactions when drinking grapefruit juice.

## Figures and Tables

**Table 1:** Precision and accuracy for tadalafil of QC samples in rat plasma (*n* = 6).

Added (ng/mL)	Intraday			Interday		
	Found (ng/mL)	RSD (%)	RE (%)	Found (ng/mL)	RSD (%)	RE (%)
25	25.97 ± 1.22	4.69	3.88	24.04 ± 1.99	8.28	−2.00
1000	483.99 ± 15.84	3.27	2.50	493.97 ± 31.09	6.29	0.30
1500	1479.15 ± 67.11	4.54	1.20	1539.65 ± 74.87	4.86	−1.53

**Table 2:** Recovery of tadalafil from rat plasma after extraction (*n* = 6).

Analytes	Added (ng/mL)	Recovery (%)	RSD (%)
Tadalafil	25	78.91 ± 2.42	3.07
	500	82.57 ± 2.32	2.81

IS	1500	81.21 ± 2.13	2.63
	400	83.35 ± 4.13	3.21

**Table 3:** Stock solution stability tests for the determination of tadalafil in rat plasma (*n* = 6).

Storage conditions	Concentration added (ng/mL)	Tadalafil	
		RSD (%)	RE (%)
Ambient, 24 h	25	4.19	2.3
	500	2.30	3.1
	1500	1.12	2.7

Three freeze-thaw	25	2.34	−2.4
	500	2.17	3.7
	1500	1.43	−5.4

−80°C, 4 weeks	25	4.03	3.9
	500	2.81	−2.7
	1500	1.21	3.1

**Table 4:** The main pharmacokinetic parameters of tadalafil after oral administration of 5 mg/kg tadalafil in rat plasma (means ± SD, *n* = 6).

Parameters	Group A (tadalafil + grapefruit juice)	Group B (tadalafil)
*t*_1/2_ (h)	11.70 ± 1.47	9.28 ± 2.07
*T*_max_ (h)	1.25 ± 0.27	1.25 ± 0.27
*C*_max_ (ng/mL)	1271.60 ± 179.31	725.89 ± 161.59
AUC_(0-*t*)_ (ng·h/mL)	9586.52 ± 2048.81	7399.61 ± 696.85
AUC_(0-∞)_ (ng·h/mL)	10639.43 ± 2235.94	7995.50 ± 707.23
CL_z_/F (L/h/kg)	0.50 ± 0.14	0.63 ± 0.06
Vd_z_/F (L/kg)	8.42 ± 2.96	8.48 ± 2.25

## Data Availability

The data used to support the findings of this study are available from the corresponding author upon request.
